# Elimination of wild-type *P53* mRNA in glioblastomas showing heterozygous mutations of *P53*

**DOI:** 10.1038/sj.bjc.6604258

**Published:** 2008-03-18

**Authors:** M Szybka, I Zawlik, D Kulczycka, E Golanska, E Jesien, D Kupnicka, R Stawski, S Piaskowski, E Bieniek, M Zakrzewska, R Kordek, P P Liberski, P Rieske

**Affiliations:** 1 Department of Pathology, Medical University of Lodz, Paderewskiego 4, Lodz 93-509, Poland; 2 Department of Molecular Pathology and Neuropathology, Medical University of Lodz, Czechoslowacka 8/10, Lodz 92-216, Poland; 3 Department of Radiology, Medical University of Lodz, Czechoslowacka 8/10, Lodz 92-216, Poland

**Keywords:** P53, glioblastoma, methylation, mRNA

## Abstract

We screened 50 glioblastomas for *P53* mutations. Five glioblastomas showed heterozygous mutations, while three were putatively heterozygous. Six of these eight glioblastomas showed elimination of wild-type *P53* mRNA. These results strongly suggest that some sort of mechanism(s) favouring mutated over wild-type *P53* mRNA exists in glioblastoma cells with heterozygous mutations of this gene.

A majority of tumour suppressor genes present homozygous or hemizygous mutations (Sherr, 2004). Intriguingly, in the *P53* gene, heterozygous mutations have also been detected. Typical mutations of this gene are of the missense type, leading to P53 protein gain of function (Nigro *et al*, 1989; Dittmer *et al*, 1993). However, the effects of at least some heterozygous mutations cannot be explained only by the gain of *P53* function (Park *et al*, 1994). In case of *P53* mutations such as R249S or R273H, at least three mutated monomers per tetramer appeared to be required to inactivate the transactivation of MDM2 and p21 CIP1/WAF1 promoters (Chan *et al*, 2004). In case of R280T mutations, heterotetramers consisting even of three mutated and one wild-type P53 monomer showed partially but not completely abolished activity compared to P53 homotetramers consisting of wild-type monomers only (Sun *et al*, 1993). In this context, the occurrence of heterozygous mutations of *P53* remains enigmatic, leading to a question of whether mechanisms other than *P53* mutations or deletions are involved in the elimination of the wild-type P53 protein. Several nongenomic mechanisms of protein elimination or aberration have been described, including processes operating at the level of transcription (e.g., methylation) or translation (e.g., miRNA) (Voorhoeve *et al*, 2006; Watanabe *et al*, 2007). We examined whether glioblastoma cells with heterozygous mutations of *P53* contained a mixture of wild-type and mutated *P53* mRNA, or predominantly the mutated *P53* mRNA. Additionally, we also checked the methylation status of the *P53* promoter.

## MATERIALS AND METHODS

### Tumour samples

The study included 50 cases of glioblastoma, diagnosed at Department of Pathology, Medical University of Lodz, according to the World Health Organization criteria for classification of brain tumours (Louis *et al*, 2007). The group consisted of 25 females and 25 males, aged from 15 to 76 years (median 59.5).

### DNA and RNA isolation

The investigations were performed using snap-frozen tissues stored at −80°. DNA was isolated from tumour tissues and blood samples from each patient. DNA and RNA were coextracted by means of Macherey-Nagel DNA/RNA purification kit. RNA samples were treated with DNAase. RNA and DNA concentrations were measured spectrophotometrically. In all 100 ng of total RNA was reverse-transcribed into single-stranded cDNA in a final volume of 40 μl containing 50 mM DTT, 1.5 *μ*g oligo(dT), 0.5 mM dNTP, 40 units RNase inhibitor and 200 units M-MLV reverse transcriptase (Promega).

### Loss of heterozygosity and microsatellite instability analyses

Loss of heterozygosity (LOH) and microsatellite instability (MSI) analyses were performed using paired tumour specimens and corresponding peripheral blood samples, to recognise tumour samples with minimal contamination by normal cells. The following LOH and MSI markers were used: D1S2734, D1S197, D1S162, D1S156, D9S319, D9S319, D9S162, D10S587, D10S1267, D17S1828, AFM119, BAT25, BAT26, BAT40. Forward primers were 5′ end fluorescence-labeled. PCR was performed in thermocycling conditions individually established for each pair of primers. PCR products were denatured and gel electrophoresis in LiCor automatic sequencer system was applied to the separation and analysis of PCR-generated alleles.

### *P53* DNA and cDNA sequencing

Exons 5–8 of the *P53* gene were amplified by PCR as described before and sequenced using the dideoxy termination method and SequiTherm Excel DNA Sequencing Kit (Epicentre Technologies) (Zakrzewska *et al*, 2005).

The primers used for the PCR amplification of cDNA sequences were: *p53*: 5′-GTGCAGCTGTGGGTTGATT-3′ (sense) and 5′ GCAGTGCTCGCTTAGTGCTC-3′ (antisense); annealing temperature was 53°C. The sequencing primers were: *p53* exon 5–8: 5′-GCCATCTACAAGCAGTCACA-3′ (sense), and *p53* exon 8–5: 5′-CCCTTTCTTGCGGAGATTCT-3′ (antisense); annealing temperature was 55°C. LiCor automatic sequencer system was applied to the separation and analysis of PCR-sequencing products.

To verify the results, a semi-quantitative densitometric analysis was performed in which wild and mutated band intensity was estimated, and then compared to a neighbouring band in the same sequencing lane for reference.

### Methylation-specific PCR (MSP)

Sodium bisulphite modification of genomic DNA was performed using the CpGenome Universal DNA Modification Kit (Chemicon International, Temecula, CA, USA). CpGenome Universal Methylated DNA (Chemicon International) was used as a methylation-positive control for the methylated *P53* promoter, and DNA from peripheral blood leukocytes was used as the control for unmethylated alleles of *P53*. The MSP was performed as previously described (Amatya *et al*, 2005).

## RESULTS

**Figure 1:**
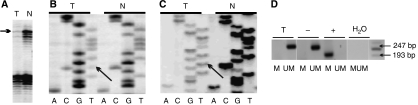
Molecular analyses of glioblastomas. (**A**) example of LOH analysis showing a minimal contamination of the tumour sample with the normal cells (case 4; only a trace of the lost allele is observed in the tumour sample). The lost allele is marked with an arrow. (**B**, **C**) *P53*-sequencing results. The mutated nucleotide (*p53* Exon 8; codon 273; CGT>TGT; Arg>Cys) is marked with arrows. (**B**) genomic DNA sequencing (case 3): C and T nucleotides are both detected, representing a heterozygous mutation. (**C**) cDNA sequencing (case 3): no wildtype, only mutated mRNA is detected. (**D**) MSP result representing a lack of P53 promoter methylation (case 3). T, tumour sample; N, a corresponding normal tissue (blood); −, negative control; +, positive control; M, methylated; UM, unmethylated.

Genomic DNA and cDNA obtained from fifty glioblastoma samples were sequenced for *P53* mutations. Mutations of *P53* were detected in 16 cases, eight of these being heterozygous (showing a weak mutated band or a mutated band as strong as the wild band; Figure 1B). Five of these eight cases were indeed confirmed as heterozygous when LOH and MSI analyses showed no or negligible contamination of the tested samples by normal cells (cases 1–5, Table 1; Figure 1A). In three additional cases (6–8, Table 1), sequencing results suggested heterozygous mutations of *P53*. However, we could not exclude a possible contamination of the tumour specimens with normal cells in this instance because no LOH/MSI was detected in them. We defined these cases as presenting putative heterozygous mutations of *P53*. In six cases (including heterozygous as well as putative heterozygous mutations), cDNA sequencing revealed a decreased amount, or lack of, the wild (nonmutated) template when compared to the genomic DNA sequencing (cases 1–4, 6–7, Table 1; Figure 1C). A densitometric analysis of the wild and mutated bands confirmed the above observations (data not shown).

MS-PCR revealed *P53* promoter methylation (Figure 1D) in only three cases. One had a heterozygous mutation, while another had a putative heterozygous mutation of the *P53*. However, in both of these cases no wild-type cDNA template was detected (case 4 and 7). The third case also presented a heterozygous mutation of *P53 –* but without any decrease in the amount of wild-type cDNA template – as shown by cDNA *vs* genomic DNA sequencing (case 5, Table 1).

## DISCUSSION

Heterozygous mutations of *P53* have been widely described (Dittmer *et al*, 1993). In this study we show that a majority of glioblastomas presenting heterozygous mutations of *P53* gene presented no wild-type *P53* mRNA. These results strongly suggest that glioblastoma cells may have the ability to develop a mechanism(s) which would allow for either (1) the silencing of wild-type *P53* transcription, (2) the degradation of wild-type *P53* mRNA, or (3) the selective overproduction of mutated *P53* mRNA. Moreover, our results show that heterozygous mutations of *P53* gene, elimination of wild-type *P53* mRNA, or selective production of mutated mRNA can occur during glioblastoma tumorigenesis. An extremely important question thus arises – that is, ‘what mechanism(s) is (are) responsible for favouring the mutated *P53* mRNA over the wild-type ones?’

A methylation of DNA regulatory elements – mainly promoters, is one of the mechanisms of tumour suppressor genes silencing (Watanabe *et al*, 2007). It was shown that a similar proportion of gliomas with and without *P53* mutation present *P53* promoter methylation (Amatya *et al*, 2005). Indeed, the lack of wild-type mRNA – despite the presence of wild-type DNA observed by us, could be explained by a selective methylation of DNA regulatory element of nonmutated *P53* allele. The primers we used in methylation-specific PCR have already been successfully used by the Ohgaki group in analysing *P53* promoter methylation in gliomas (Amatya *et al*, 2005). Nevertheless, we observed that no *P53* promoter methylation was detectable with this set of primers in the majority of cases analysed in this study. Collectively, these results suggest that identification of mechanism(s) responsible for the elimination of wild-type *P53* mRNA requires more research. Obviously, the elimination of wild-type P53 may have resulted from a mechanism other than epigenetic changes of *P53* DNA regulatory elements. Nonetheless, uncovering of this mechanism can be very important for the development of new anti-tumour therapeutics.

In conclusion, we show in this article that glioblastomas presenting heterozygous mutations of *P53* employ some sort of mechanism(s) to positively select mutated *P53* mRNA. We offer a relatively easy procedure for determining whether similar situations also occur in other cancers. The precise mechanism(s) for favouring the mutated type of *P53* mRNA – however, still remains to be discovered.

## Figures and Tables

**Table 1:** DNA *vs* cDNA sequencing in glioblastomas showing *P53* mutations

**Case No**	**Mutation**	**DNA sequencing**	**cDNA sequencing**	**LOH and/or MSI analysis**	**MS-PCR**
*Heterozygous mutations*					
1	273 Arg-His ex8	Domination of normal template	Only mutated template visible	Cells without MSI not detected	UM
2	234 Tyr-His ex7	Domination of normal template	Only mutated template visible	Cells without MSI not detected	UM
3	273 Arg-Cys ex8	Domination of normal template	Only mutated template visible	Cells without LOH not detected	UM
4	175 Arg-His ex5	Domination of normal template	Domination of mutated template	Minimal contamination with cells without LOH	M
5	282 Arg-Trp ex8	Domination of normal template	Domination of normal template	Cells without LOH not detected	M
					
*Putative heterozygous mutations*					
6	190 Pro-Ser ex6	Mutated and wild type template equally presented	Only mutated template visible	No LOH, no MSI detectable	UM
7	237 Met-Ile ex7	Domination of normal template	Only mutated template visible	No LOH, no MSI detectable	M
8	273 Arg-His ex8	Domination of normal template	Domination of normal template	No LOH, no MSI detectable	UM

LOH=Loss of heterozygosity; M=methylated; MIS=microsatellite instability; MS-PCR=methylation-specific PCR; UM=unmethylated.
